# A novel *KDM5C* mutation associated with intellectual disability: molecular mechanisms and clinical implications

**DOI:** 10.1186/s13052-025-01887-y

**Published:** 2025-02-14

**Authors:** Yunlong Meng, Xinyao Wang, Kangyu Liu, Xingkun Tang, Haining Li, Jianjun Chen, Zilin Zhong

**Affiliations:** 1 Shanghai Key Laboratory of Anesthesiology and Brain Functional Modulation, Clinical Research Center for Anesthesiology and Perioperative Medicine, Translational Research Institute of Brain and Brain-Like Intelligence, Department of Pediatrics, Shanghai Fourth People’s Hospital, School of Medicine, Tongji University, Shanghai, 200434 China; 2 Institute of Medical Genetics, Department of Child, Adolescent and Maternal Health, School of Public Health and General Medicine, School of Medicine, Tongji University, Shanghai, 200092 China; 3 Tongji University School of Medicine, 500 Zhennan Road Putuo District, Shanghai, 200331 China

**Keywords:** *KDM5C*, Gene mutation, ID, Genetic analysis, Nonsense-mediated mRNA decay

## Abstract

**Background:**

Among the disease-causing genes associated with X-linked intellectual disability (XLID), *KDM5C* is one of the most frequently mutated ones. *KDM5C* is a widely expressed gene that is most highly expressed in the brain. *KDM5C* modulates the transcriptional activity of genes through demethylation of H3K4, thereby regulating neural development and normal function. We identified a gene from a Chinese family and found that a nonsense mutation of *KDM5C* was co-segregated with the intellectual disability (ID).

**Methods:**

The candidate mutant genes of patients with ID phenotype were screened by Whole Exome Sequencing (WES), and DNA Sanger sequencing was performed for genetic analysis. Pathogenicity prediction tools were used to evaluate the pathogenicity of new mutations. The fusion plasmid was constructed and transfected into the cells, and the changes of mRNA and protein levels of the mutants were detected by semi-qRT-PCR and Western Blot, and the subcellular localization changes of mutant proteins were detected by Immunofluorescence technique.

**Result:**

The nonsense mutation in *KDM5C* (c.2785 C > T, p. R929X) was identified by whole exome sequencing (WES) and confirmed by Sanger sequencing, resulting in a truncated protein. The mutation was determined by pathogenicity prediction tool able to find non-sense mediated mRNA decay (NMD). Semi-qRT-PCR and Western Blot showed that the mRNA levels of the mutant gene were down-regulated, while the protein level was up-regulated. Additionally, the subcellular localization of the mutant protein changed.

**Conclusions:**

The *KDM5C* mutation found in our study leads to changes in protein levels through NMD and/or protein degradation, and produces residues lacking nuclear localization, thus altering the subcellular localization of the protein. These results may lead to changes in the expression of *KDM5C* target genes, ultimately contributing to the clinical phenotype observed in the patients.

**Supplementary Information:**

The online version contains supplementary material available at 10.1186/s13052-025-01887-y.

## Introduction

For a long time, X-linked gene defects have been considered as important pathogenic causes of intellectual disability (ID). This is due to the ID related genes located on the X chromosome, which are more significantly expressed in the central nervous system than those on the autosomal chromosome [1]. Among the pathogenic genes associated with XLID, *KDM5C* is one of the most frequently mutated, with an estimated involvement in 0.7-2.8% of XLID cases [2, 3].

*KDM5C* (formerly known as JARID1C or SMCX) is expressed in tissues including brain, heart, skeletal muscle, liver, pancreas and lungs, but at the highest level in the brain [1, 4, 5]. *KDM5C* is located at Xp11.22, containing 26 exons and encoding a 1560 aa protein, which is a member of KDM5 family [6, 7].

Widespread expression of *Kdm5c* has been detected in whole brain slices of adult mice [8], indicating that *KDM5C* is crucial for neural development and normal function. In HeLa cells, *KDM5C* is co-located with the transcriptional repressor REST at the promoters of a set of REST target genes, suggesting that the loss of *KDM5C* activity impairs REST-mediated neuronal gene regulation [9]. In zebrafish, down-regulation of *kdm5c* leads to an increase in nerve cell death and a decrease in the total length of dendrites [10]. The expression of specific neuronal genes is repressed in stem cells and non-neuronal tissues, partly due to the demethylation of H3K4 provided by *KDM5C* in the promoter sequences [9]. In their research report on induced pluripotent stem cells from human patients and *Kdm5c* gene knockout mice, Violetta Karwacki-Neisius and her colleagues determined that *KDM5C* directly controls the output of WNT (**wingless-related integration site)** to regulate the timely transition from primary progenitor cells to intermediate progenitor cells, thereby regulating neurogenesis to ensure that neurodevelopment occurs on an appropriate timescale [11].

In this study, we identified a molecular defect in a Chinese family by whole exome sequencing (WES) and Sanger sequencing. We found a variation of *KDM5C* (c. 2785 C > T). The mutation of *KDM5C* was co-segregated with the phenotype of ID among patients. The mutation produces a premature termination codon (PTC) at position 929 of the protein, resulting in reduced transcription levels and translation expression levels of the *KDM5C*. In addition, the mutant is missing a PHD domain and nuclear localization residue, resulting in a failure of nuclear localization of the protein. Performing functional analysis of these variants in vitro and further determining the relationship between genetic variants and diseases is of great significance for deciphering the pathogenic mechanism of diseases caused by variants, and providing a basis for prenatal diagnosis and genetic counseling.

## Methods

### Patients and clinical data

We recruited a Chinese family with ID. This study was approved by the Institutional Review Board of Tongji University School of Medicine (Shanghai, China) and conformed to the tenets of the Declaration of Helsinki. About 5 ml of peripheral venous blood samples were collected in vacutainer tubes (Becton Dickinson [BD], Sunnyvale, CA, USA) containing EDTA from available patients and their unaffected family members. Genomic DNA was extracted using DNA extraction kits (Tiangen Biotech Co., Ltd., Beijing, China).

### Variant screening and sequencing

Candidate variants in family was screened using whole-exome sequencing (WES) by Genesky Bio-Tech Co., Ltd., (Shanghai, China). To confirm whether the disease phenotype was co-segregated with the candidate gene in the family, DNA samples from the members of the family were amplified using polymerase chain reaction (PCR). The following primers were used to screen for the *KDM5C* mutation: *KDM5C*-EX17F: gtgggacaaggttccatctg; *KDM5C*-EX17R: ccactcaactttgatgtttgga. Finally, the PCR products were validated by Sanger sequencing using an ABI3730 Automated Sequencer (Applied Biosystems; Thermo Fisher Scientific, Inc., USA), and compared with the *KCM5D*(NM_004187) reference sequence in the National Center for Biotechnology Information database (http://www.ncbi.nlm.nih.gov/).

### Plasmid construction

The cDNA sequence plasmid containing *KDM5C* was purchased from BIO-RESEARCH INNOVATION CENTER SUZHOU (Suzhou, China) and the corresponding wild type and mutant coding sequences were amplified by PCR. The amplified product was cloned into pEGFP-C1 vector (XhoI and BamHI endonuclease 37 ℃ for 4 h) by pEASY^®^-Uni Seamless Cloning and Assembly Kit (TransGen Biotech, China) to obtain *KDM5C* wild-type plasmid (pEGFP-*KDM5C*_WT) and variant plasmid (pEGFP-*KDM5C*_MT) linked to EGFPC terminal. The primers used for amplification are listed in supplementary Table 1. The wild type and variant plasmids were sequenced to verify the inserted sequence and target mutation.

### Cell culture and transfection

HeLa and HEK293T cells were cultured in high-glucose Dulbecco’s modified Eagle medium (DMEM, Invitrogen; Thermo Fisher Scientific, Inc.) with 10% fetal bovine serum (FBS; Invitrogen; Thermo Fisher Scientific, Inc.) in a humidified atmosphere containing 5% CO_2_ at 37 °C. According to the manufacturer’s plan, Lipo8000™ Transfection Reagent (Beyotime Institute of Biotechnology, China) was used for transfection.

### Gene mRNA analysis

HeLa cells were collected 36 h after transfection with wild-type and variant plasmids. Total RNA of cell was extracted by Trizol (Tiangen, China). The total RNA (OD260/280 ≈ 2.0) of about 1 µg was then reverse-transcribed into first-strand cDNA with FastKing one-step RT-PCR kit (Tiangen, China) and then stored at -20 ℃ until use. The primers used for amplification are listed in supplementary Table 1.

### Immunofluorescence

HeLa cells were plated onto coverslips in 12-well plates and seeded at 3 × 104 cells per well in DMEM with 10% FBS. After 36 h transfection, the culture medium has been carefully aspirated. Cells were washed three times with phosphate-buffered saline (PBS) to remove phenol red and any residual medium. Cells were fixed by adding 4% paraformaldehyde (PFA) in PBS and incubated at room temperature for 20 minutes. Cells were washed three times with PBS to remove residual paraformaldehyde. The nuclei were stained with DAPI dye (0.5 µg/m1) for 1 min. Cells were washed three times with PBS to remove excess DAPI. The location of *KDM5C* wild-type and mutant proteins were detected by confocal fluorescent microscopy at 40× magnifications.

### Western blot

Following 36 h transfection with the wild-type and variant plasmids into HEK293T cells, cells were lysed on ice using radio immunoprecipitation assay lysis buffer (P0013B; Beyotime Institute of Biotechnology, China) containing proteinase inhibitors. Total proteins were extracted from the supernatant after centrifugal at 12,000 rpm for 15 min at 4 ℃. For western blotting, total protein was quantified using a bicinchoninic acid assay (Beyotime Institute of Biotechnology, China). Protein samples were fully denatured with SDS–PAGE protein loading buffer (Beyotime Institute of Biotechnology, China), followed by resolving using 8% SDS–PAGE and transferring to polyvinylidene fluoride membranes. After blocking with 5% non-fat milk at 25 ℃ for 1 h and TBST washing for three times, the EGFP-KDM5C recombinant proteins were detected with a 1:1000 dilution anti-EGFP and anti-ACTB antibody monoclonal antibody (produced from mouse, Sangon Biotech Co., Ltd., China) at 4 ℃ overnight. The secondary antibody (Goat Anti-Mouse IgG; Sangon Biotech Co., Ltd., China) was used at a dilution of 1:5000. Membranes were visualized using chemiluminescent Western Blotting Substrate (Bio-Rad Laboratories, Inc., USA) with Tanon Imaging System (Tanon Science & Technology Co. Ltd., China).

### Statistical analysis

The blot bands and data were analyzed using ImageJ (version 1.8.0) and GraphPad Prism software (version 8.0.2), in which the expression levels of the target protein were normalized relative to ACTB expression, and the target mRNA were normalized relative to *GAPDH* expression. All the in vitro experiments were repeated at least three times. An unpaired t-test was employed for the analysis, and a significant level of *P* < 0.05 was used to determine statistical significance.

## Results

### Case presentation

Herein, we describe clinical and genetic findings from a China family co-segregating a nonsense mutation (c.2785 C > T)in exon 19 of *KDM5C* gene with ID phenotype. The mutation produces a premature termination codon at position 929 of the protein (p.R929X) before the PHD domain, resulting in reduced *KDM5C* mRNA expression levels.

**Fig. 1:**
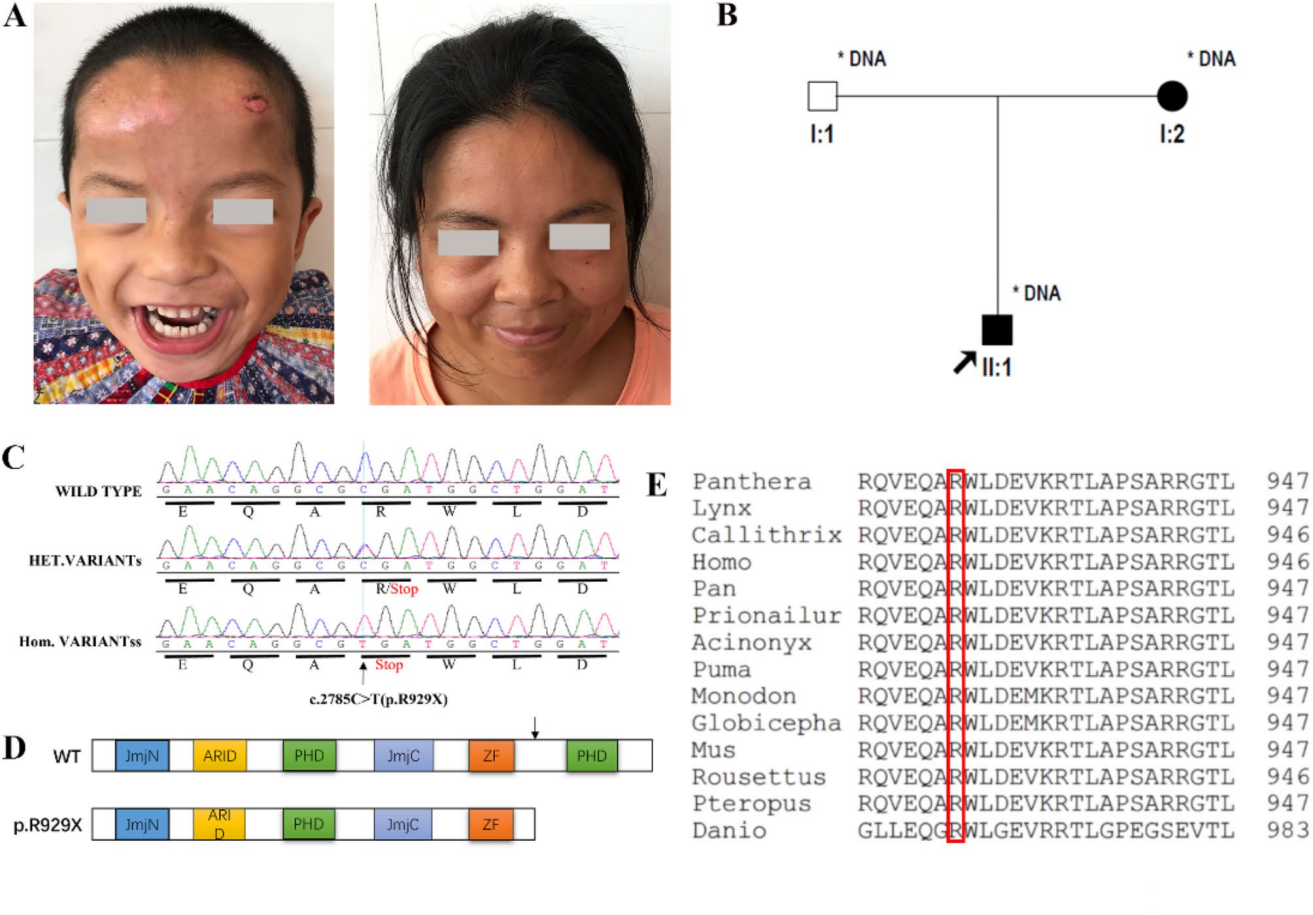
The *KDM5C* mutation was identified in the genetic pedigree of the family. (**A**) Frontal views of the proband and the mother in the family; (**B**) Pedigrees in the families with ID. Available DNA samples were marked with asterisk; Arrows represent proband; Black fillings represent diseased individuals; The box represents the male, the circle represents the female. (**C**) Sequence of *KDM5C* mutations identified in the families. (**D**) Schematic representation of KDM5C WT and mutated proteins. JmN = jumonji-N domain; ARID = AT-rich interacting domain; PHD = plant homeodomain box domain; JmjC = jumonji-C catalytic domain; ZF = zinc finger domain; WT, wild-type. (**E**) Conserved analysis of p.R929 in different species

A Chinese family consisting of the proband, a mother with mild ID and an asymptomatic father is described here. The proband is a 14 young boy, the child of a young and unrelated couple. The patient was delivered via vagina and there were no complications in pregnancy. The proband suffered from severe ID, short stature, epilepsy (treatment with sodium valproate), obvious expressive language disorder (during the interactive communication sessions with the proband), relationship disorder, aggressive behavior, hyperactivity and learning difficulties and self-harm tendencies (e.g., forehead injuries from headbanging Fig. 1A). The mother displayed clinical signs indicating mild intellectual disability and short stature (Fig. 1A). From the genetic pedigree of the family, it is known that the pathogenic gene is inherited from the mother to the patient, and it can be inferred that the gene is autosomal dominant or X-chromosome dominant mode of inheritance (Fig. 1B).

### Mutation analysis and cosegregation analysis

We tested the members of the family for WES. The results showed that the proband (individual II:1) showed a hemizygous mutation in exon 19 of *KDM5C* (c. 2785 C > T), which was not found in the unaffected father I:1 (individual I:1), and the mother (individual I:2) was a heterozygous carrier of this mutation. This was confirmed in the Sanger sequencing assay of PCR products derived from DNA extracted from blood samples of members of the family (Fig. 1C). *KDM5C* sequencing from available members of the family proved that the truncating mutation co-segregated with the ID phenotype, since the variant was present in all genotyped individuals with ID (Fig. 1B; affected mother, I:2, and proband, II:1) and absent in unaffected males (Fig. 1B; unaffected father, I:1). This mutation has not been reported in the literature and not found in ExAC, 1000G, or gnomAD, which indicates that they may be the potential pathogenic variants instead of genetic polymorphism. The mutation causes the production of a premature termination codon at the 929(p.R929X) position of the protein (Fig. 1D). We utilized mutation prediction tools like Mutation Taster(A), Inter Var (Pathogenic), which showed that the mutation was harmful. In addition, based on the cross-species alignment analysis of the amino acid sequence of the mutation site, we found that arginine 929 was highly conserved in different species (Fig. 1E).

### Expression of wild type and mutation *KDM5C*

**Fig. 2:**
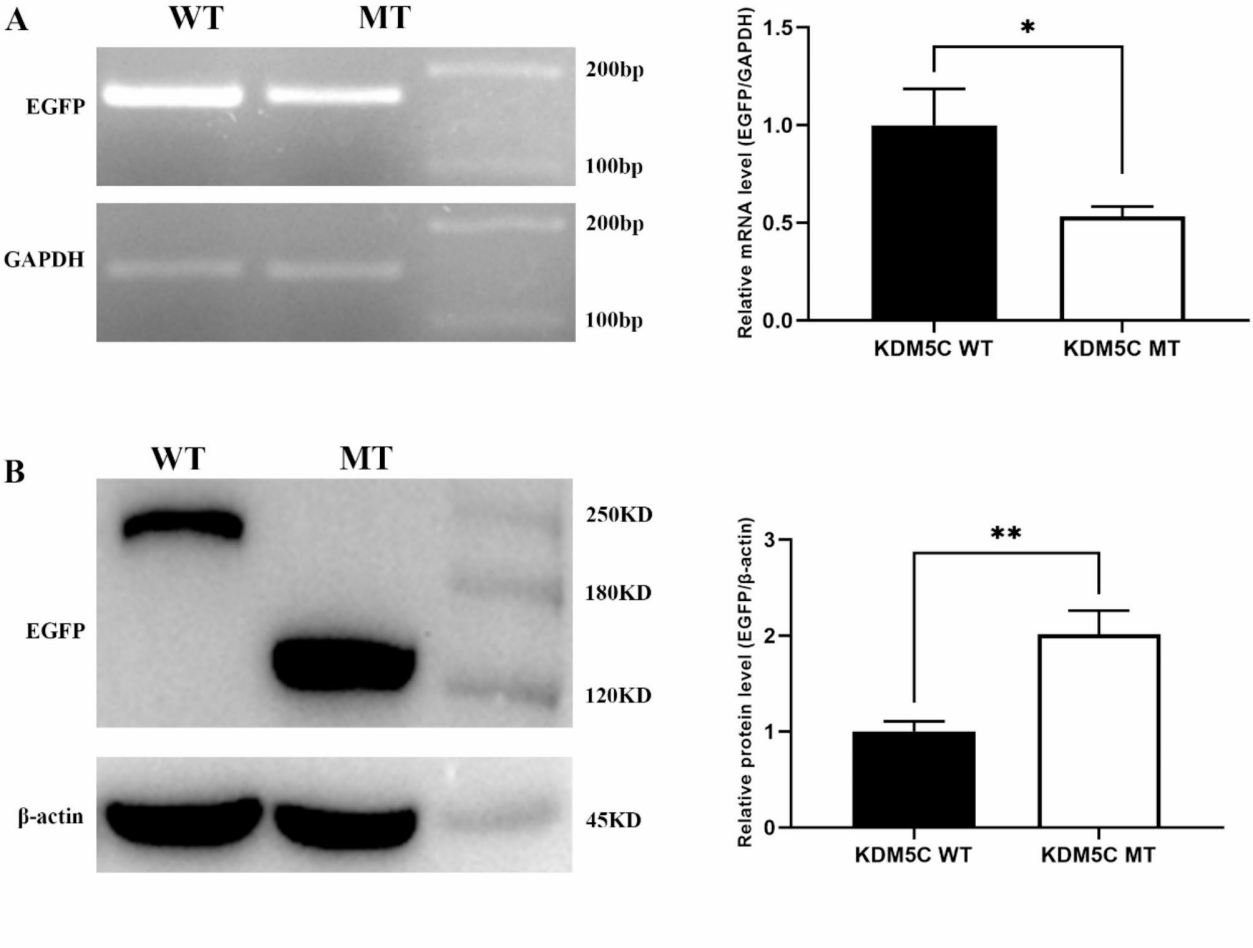
The mRNA and protein expression levels of *KDM5C* were detected after transfection of WT and MT *KDM5C* and *EGFP* fused plasmids. (**A**) Detection of *KDM5C* mRNA expression level by semi-quantitative PCR (represented by *EGFP* expression). (**B**) Detection of KDM5C protein expression by Western Blot (expressed as EGFP). WT, wild-type; MT, mutation

We used Mutation Taster to predict that this mutation would lead to NMD. When a premature termination codon is introduced, it leads to nonsense-mediated decay of mRNA, resulting in very little protein production [12]. It has been demonstrated that nonsense mutations in *KDM5C* (c.3864G > A, c.2080 C > T) lead to a significant reduction in *KDM5C* mRNA levels [5]. To test whether the nonsense c. 2785 C > T mutation also affects *KDM5C* mRNA levels, we used semi-qRT-PCR to detect the transcriptional expression of the plasmid carrying the mutation site in cells. We found that the mRNA expression level of the mutant gene was significantly lower than that of the wild type gene (Fig. 2. A). In addition, we also tested its protein expression level by Western Blot. The results showed that the expression level of mutant was significantly up-regulated than that of wild type (Fig. 2. B).

### Subcellular localization

**Fig. 3:**
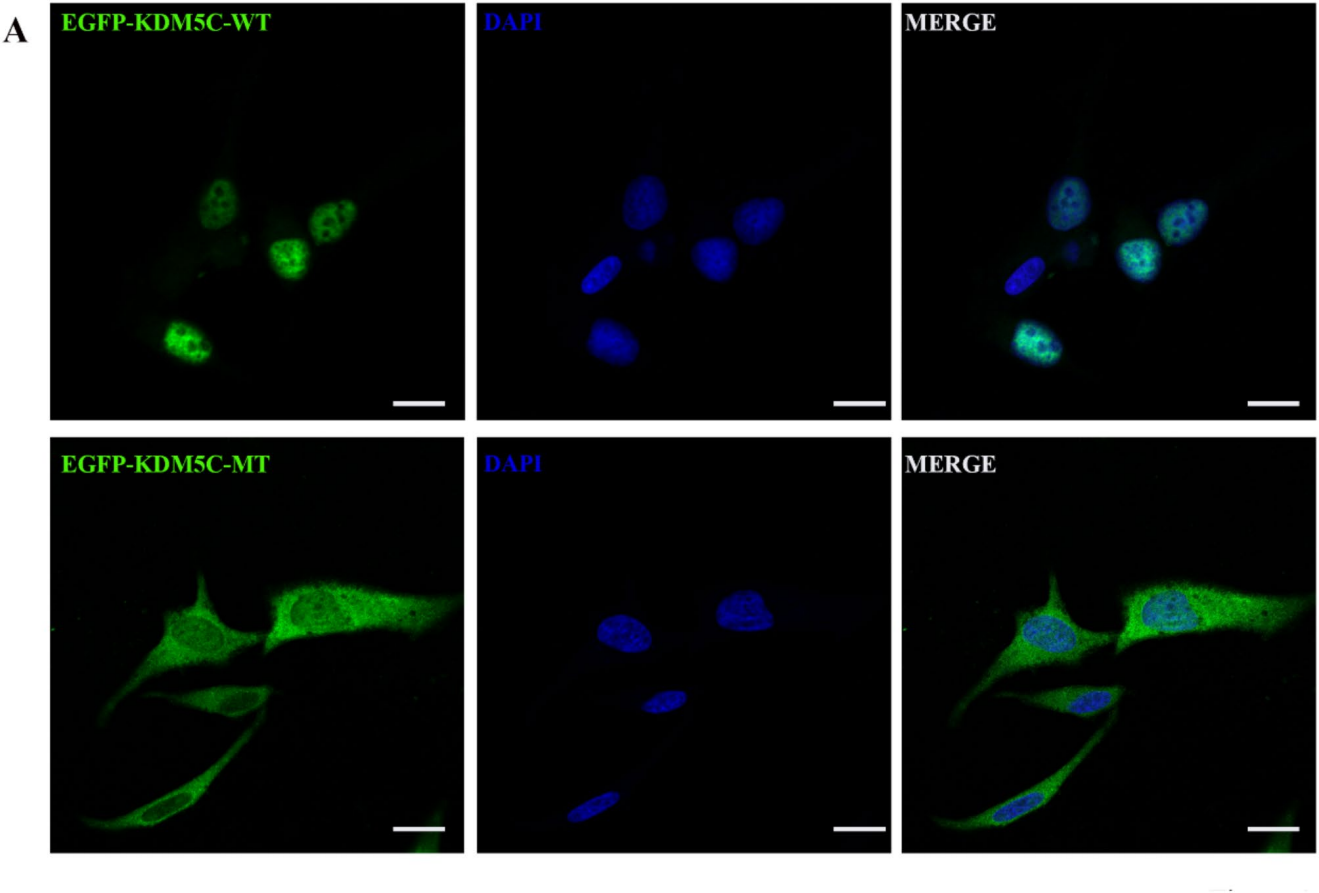
Subcellular localization of the KDM5C WT and MT proteins. EGFP is fused with the KDM5C WT or MT proteins to visualize their subcellular localization (shown in green). DAPI is used to stain nuclear DNA (shown in blue). WT: wild-type, MT: mutant. Scale bar: 20 μm

KDM5C specifically targets the activity of dimethylation and trimethylated lysine 4 demethylation of histone H3, and acts as a transcriptional repressor to regulate the activity of target genes, such as directly controlling the expression of WNT to regulate the timely transformation of primary progenitor cells to intermediate progenitor cells, thereby regulating neurogenesis [4, 9–11, 13–15]. Therefore, the correct subcellular localization is very critical. We transiently introduced the fusion plasmid into HeLa cells and used immunofluorescence technique to observe the difference of subcellular localization between wild type and mutant type. Our experimental results show that wild-type KDM5C is located in the nucleus as a transcription factor, while mutant KDM5C is located in the cytoplasm (Fig. 3A). This indicates that the p.R929X mutation site changes the role of KDM5C entering the nucleus and acting as a transcription factor.

## Discussion

Most *KDM5C* variant phenotypes are observed in males, while some heterozygous females remain asymptomatic, though a portion may present with mild ID and spasticity [14–19]. The two patients we reported were a mother (I:2) and her son (II:1). The hemizygous affected male patient, exhibited severe ID, short stature, epilepsy, expressive language disorder, social difficulties, aggressive behavior, hyperactivity and learning difficulties and self-harm tendencies. The female patient, a heterozygous carrier, presented with mild ID and short stature. Compared to males with hemizygous mutations, female heterozygous carriers are typically asymptomatic or mildly affected, likely due to the X-chromosomal inactivation skewing. Disruption of the *Kdm5c* gene in male mice mimics XLID-related cognitive abnormalities, while female *Kdm5c* knockout mice show milder impairments, primarily memory deficits and learning disabilities [20, 21]. We analyzed the species conservation of the amino acids at this mutation site (p.R929X), and found that is highly conserved, suggesting that this nonsense mutation could result in significant functional impairment.

NMD is a eukaryotic mRNA quality control mechanism that prevents disease by degrading mutant mRNAs with premature stop codons, thus avoiding the production of harmful truncated proteins [12, 22–24]. Wu et al. reported that the *KDM5C* mutation (p.E1131Afs) involved a small insertion deletion, leading to protein truncation [25]. Compared with WT *KDM5C*, the mRNA level of the *K*DM5C mutation (p.E1131Afs) remained unchanged, but the protein level was significantly reduced. Poeta et al. reported that the mRNA levels of mutated *KDM5C* (p.W1288X and p.W534Gfs*15) were reduced, functioning as deletion mutations since they could not produce functional proteins [26]. In our study, the *KDM5C* nonsense mutation (p.R929X) contains a premature termination codon (PTC). We analyzed the mRNA and protein levels of *KDM5C* and found that the mutant mRNA was reduced compared to the wild type. However, the upregulation of the mutant protein suggests that the mutation may impact protein stability. Xiao et al. reported that TRIM11 is a ubiquitin E3 ligase for KDM5C, and that TRIM11 ubiquitinates the intact KDM5C fragment (171-1560aa), promoting its degradation via the proteasome [27]. However, the mutant protein (p.R929X) lacks the complete 171-1560aa fragment, preventing its degradation by TRIM11-mediated ubiquitination, which may explain the elevated levels of the mutant protein. The *KDM5C* mutation leads to abnormally elevated protein levels, which may further exacerbate the patient’s neurological damage, resulting in severe symptoms such as epilepsy and cognitive decline. Therefore, future studies should focus on exploring the impact of this mutation on protein degradation pathways and validate these findings in mouse or zebrafish models.

KDM5C includes catalytic JmjC domain, a zinc finger-C5HC2 domain, JmjN domain responsible for protein stability, Bright/ARID responsible for DNA binding domain, and two plant homologous domains (PhD) for histone binding [5, 28]. The mutant we reported produced the PTC at position 929 (p.R929X), resulting in a truncated protein with a deletion of the C-terminal PhD domain, which affected its binding to histones. In addition, the truncated fragment lacks a hypothetical nuclear localization signal (residue 1448–1489) [29]. Our immunofluorescence staining experimental results show that this mutant affects the localization of KDM5C proteins to the nucleus.

Pathogenic variants of *KDM5C* may contribute to the neurodevelopmental features of Claes-Jensen syndrome by affecting multiple key transcriptional programs [19]. *KDM5C* gene silencing leads to down-regulation of *SCN2A*, *CACNA1B*; *CACNA1H*; *SCL4A3*, *SLC18A1*, and *SLC6A12* [9], All of these *KDM5C* target genes have been linked to neurological disorders such as epilepsy, autism or schizophrenia [30–36]. The abnormalities in the patients we reported may be related to a large number of KDM5C mutant proteins which are left in the cells, and the failure of KDM5C to locate the nucleus and bind to histone proteins, further affecting the abnormal expression of a range of target genes.

The results of testing for X-linked diseases play a crucial role in genetic counseling, providing accurate information for assessing recurrence risks and developing personalized management plans. For example, studies on *EDA* gene mutations have demonstrated that genetic testing has significant value in prenatal diagnosis, helping families make informed decisions and guiding personalized treatment [37]. Additionally, studies on other gene mutations, such as *CRLF1*, have shown how early diagnosis can significantly improve patient outcomes, particularly in the management of rare genetic diseases [38]. The *KDM5C* mutation is closely associated with severe intellectual disability, epilepsy, and abnormal behavior in patients, indicating its significant impact on neurodevelopment, especially in childhood. Early diagnosis and personalized interventions are crucial for these patients, providing essential information for genetic counseling and aiding in the assessment of recurrence risks for future family members [38–40].

In complex genetic diseases, genetic analysis helps improve diagnostic accuracy, particularly in detecting certain rare syndromes and structural defects. Genetic testing can assist in developing personalized long-term management strategies [39, 41–43]. As research into specific gene mutations, such as *KDM5C*, continues to advance, new treatment approaches may be developed in the future. Based on the research by Karwacki-Neisius et al., regulating the WNT signaling pathway may help reverse the neurodevelopmental defects caused by *KDM5C* mutations, offering new possibilities for personalized treatments [11]。.

## Conclusion

In conclusion, our study identified nonsense mutation of KDM5C was co-segregated with the intellectual disability (ID). The mutant down-regulates mRNA expression through NMD, and produces a residue that lacks nuclear localization, thus altering the subcellular localization of the protein. Additionally, the mutant leads to the accumulation of harmful protein residues. These changes may affect the expression of *KDM5C* target genes and ultimately contribute to the patient’s clinical phenotype. Our data can help in prenatal diagnosis and genetic counseling.

## Electronic supplementary material

Below is the link to the electronic supplementary material.

Supplementary Material 1

## Data Availability

The datasets used and analyzed during the current study are available from the corresponding author on the reasonable request.

## Abbreviations

XLID: X-linked intellectual disability
ID: Intellectual disability
WES: Whole Exome Sequencing
NMD: Nonsense-mediated mRNA decay
JmN: Jumonji-N domain
ARID: AT-rich interacting domain
PHD: Plant homeodomain box domain
JmjC: Jumonji-C catalytic domain
ZF: Zinc finger domain
PTC: Contain premature termination codon
