# Supplementary material for: A novel KDM5C mutation associated with intellectual disability: molecular mechanisms and clinical implications
Source: Ital J Pediatr. 2025 Feb 14;51:47. doi: 10.1186/s13052-025-01887-y (PMC11827480; doi:10.1186/s13052-025-01887-y)
Supplement: Supplementary file 1 — Supplementary Material 1 [file 13052_2025_1887_MOESM1_ESM.docx]

Supplementary table 1. Primer information for constructing plasmid of fusion protein Semi-quantitative PCR

| Plasmid construction | |
| --- | --- |
| Primer name | Primer sequence |
| KDM5C FP OL | GTACAAGTCCGGACTCAGATCTCGAGGCatggagccggggtccg |
| KDM5C RP OL | CAGTTATCTAGATCCGGTGGATCCtcacaactgttgctgaggcgg |
| KDM5C WT FP | aacaggcgCgatggctg |
| KDM5C WT RP | cagccatcGcgcctgtt |
| KDM5C MT FP | aacaggcgTgatggctg |
| KDM5C MT RP | cagccatcAcgcctgtt |
| KDM5C CAG FP | ggagccaaccttgtgCAGtgtaacacacgtccatttg |
| KDM5C CAG RP | caaatggacgtgtgttacaCTGcacaaggttggctcc |
| Semi-quantitative PCR | |
| EGFP FP | CAAGGAGGACGGCAACATC |
| EGFP RP | GTTCTGCTGGTAGTGGTCGG |
| GADPH FP | GCACCACCAACTGCTTAGC |
| GADPH RP | CATCACGCCACAGTTTCCC |
